# A high-quality genome assembly and annotation of *Thielaviopsis punctulata* DSM102798

**DOI:** 10.1038/s41597-024-03458-y

**Published:** 2024-07-09

**Authors:** Gouthaman P. Purayil, Esam Eldin Saeed, Archana M. Mathai, Khaled A. El-Tarabily, Synan F. AbuQamar

**Affiliations:** 1 Department of Biology, College of Science, United Arab Emirates University, Al Ain, 15551 United Arab Emirates; 2 Khalifa Center for Genetic Engineering and Biotechnology, United Arab Emirates University, Al Ain, 15551 United Arab Emirates

**Keywords:** Pathogens, Fungal genomics

## Abstract

Black scorch disease (BSD), caused by the fungal pathogen *Thielaviopsis punctulata* (*Tp*) DSM102798, poses a significant threat to date palm cultivation in the United Arab Emirates (UAE). In this study, Chicago and Hi-C libraries were prepared as input for the Dovetail HiRise pipeline to scaffold the genome of *Tp* DSM102798. We generated an assembly with a total length of 28.23 Mb comprising 1,256 scaffolds, and the assembly had a contig N50 of 18.56 kb, L50 of three, and a BUSCO completeness score of 98.6% for 758 orthologous genes. Annotation of this assembly produced 7,169 genes and 3,501 Gene Ontology (GO) terms. Compared to five other *Thielaviopsis* genomes, *Tp* DSM102798 exhibited the highest continuity with a cumulative size of 27.598 Mb for the first seven scaffolds, surpassing the assemblies of all examined strains. These findings offer a foundation for targeted strategies that enhance date palm resistance against BSD, and foster more sustainable and resilient agricultural systems.

## Background & Summary

Date palm (*Phoenix dactylifera* L.) is one of the oldest key fruit crop that is traditionally cultivated in arid regions of the Arabian Peninsula, Middle East and North Africa^1^, including the United Arab Emirates (UAE)^2,3^. More than 8.5 metric tons of dates are produced annually^4^, with an estimated 258,000 tons produced by 17,000 farmers in the UAE alone^5,6^. Many fungal diseases, however, wreak havoc on date palm farming and yield, resulting in significant losses in date production.

For example, Bayoud disease caused by the soil-borne fungal pathogen, *Fusarium oxysporum* f. sp. *albedinis* (*Foa*), specifically infects the roots and the vascular system of date palms, leading to widespread destruction of date palm plantations in North Africa^7^. Although *Foa* and Bayoud disease were not detected in the UAE, other *Fusarium* spp., such as *F. oxysporum* DSM106834, *F. proliferatum* DSM106835 and *F. solani* DSM106836, cause sudden decline syndrome (SDS) on date palm^8,9^. Black scorch disease (BSD, also known as Medjnoon) is a fungal disease that also affects date palms, leading to significant economic losses^10^. Disease symptoms, such as the formation of black charcoal-like lesions on leaves, inflorescence blight, and heart and bud rot, often appear on infected date palm trees^11^. Eventually, tissue necrosis, wilting, neck bending, and death of terminal buds and whole plant are associated with later stages of infection.

In 1932, Koltz first detected BSD on date palm trees, and identified *Thielaviopsis paradoxa* as the causative agent of the symptoms in the United States^11^. The same fungal pathogen was also diagnosed in Egypt on date palms in 2007^12^. Recent reports, however, identified *Thielaviopsis punctulata* (*Tp*) on date palm trees showing symptoms of BSD in Spain^13^, Egypt^14^, Qatar^15^, and Saudi Arabia^16^. In the UAE, *Tp* DSM102798 was associated with BSD of date palm^10^. This soil-borne wound pathogen can produce two types of conidia: thick-walled, oval-shaped aleuroconidia (chlamydospores) and smooth-walled, cylindric phialoconidia (endoconidia)^10^. In general, aleuroconidia are larger than phialoconidia in all *Thielaviopsis* spp.^14^. Although aleuroconidia help *Tp* adapt to extreme desert conditions for prolonged periods, phialoconidia enable the fungus to grow fast under favourable conditions.

Even though chemical pesticides are extensively used in agriculture, they do not provide a sustainable long-term solution for managing plant diseases^17–19^. Whole-genome studies, including genomics and transcriptomics, offer valuable tools for understanding the genetic basis of resistance, susceptibility, and other factors related to plant diseases^9,20,21^. Therefore, we performed highly accurate *de novo* genome sequencing and assembly of *Tp* DSM102798 using high-throughput sequencing libraries along with Hi-C for chromosome-scale scaffolding^22^. We also corrected misjoins, scaffolding uncertainty and errors in contigs by comparing with other reference genomes. Finally, we assessed the quality of Chicago and Hi-C assemblies according to the contiguity of assembled sequences (N50), completeness of conserved protein-coding genes, and Gene ontology (GO) analysis. The assembled and annotated high-quality genome of *Tp* DSM102798 not only provides genetic resources for comparative genome studies among *Thielaviopsis* spp. but also addresses the potential application of genetic-based approaches to improve sustainable date palm production.

## Methods

### Sample collection and DNA extraction

Samples of entirely dried leaves and black scorched basal parts were collected from diseased date palms from the Al-Wagan area, Abu Dhabi, UAE (latitude 24.13; longitude 55.74). The rotting tissues were sectioned into smaller pieces and used as colony starter in potato dextrose agar (PDA; Sigma Aldrich) supplemented with penicillin-streptomycin to avoid bacterial contamination. The fungus was frequently sub-cultured from the initial plates every 10–14 days until pure cultures of *Tp* were obtained.

DNA extraction was carried out on pure cultures of *Tp* grown on PDA. High molecular weight (HMW) DNA was extracted by first scraping all visible fungal material from the Petri dish, which was then transferred to a 50 ml tube containing 2 ml H_2_O. This mixture was flash-frozen to create a pellet of ~500 mg that was then ground. In the ground sample, 10 ml of cetyltrimethylammonium bromide (CTAB) and 100 µl of β-mercaptoethanol (BME) were added and incubated at 68°C for 15 minutes. After incubation, 10 µl of protease and 1 µl of RNase were added to the sample and incubated at 60°C for 30 minutes. Phenol/chloroform/isoamyl-alcohol was used to extract DNA from the cell lysate, centrifuged into a pellet, and resuspended in 200 µl Tris-EDTA (TE) buffer.

### Library preparation and sequencing

The isolated HMW DNA fragments were subjected to quality control (QC) check by measuring the concentration, the 260/280 and 260/230 ratios, and the average fragment size using pulsed-field gel electrophoresis (PFGE). After successfully passing the QC assessment, the fragments were employed in library preparation. First, Chicago libraries were prepared using ~500 ng of HMW DNA with mean fragment length = 100, which was reconstituted into chromatin *in vitro* and fixed with formaldehyde. Fixed chromatin was digested with DpnII, the 5′ overhangs filled in with biotinylated nucleotides, and then free blunt ends were ligated. After ligation, crosslinks were reversed and DNA was purified. The purified DNA was treated to remove biotin that was not internal to ligated fragments. The DNA was then sheared to ~350 bp mean length fragment size and sequencing libraries were generated using NEB Next Ultraenzymes and Illumina-compatible adapters. Biotin-containing fragments were isolated using streptavidin beads before PCR enrichment of each library. For a 1 Gb genome, it is recommended to use one library and 200 million read pairs. The Chicago sequencing library was 2213.48 times larger than the 28.2 Mb genome size of *Tp*. The Chicago libraries were then subjected to QC by sequencing 1–2 M PE, 75 bp reads on the Illumina MiSeq instrument and the reads were mapped back to the draft assembly, GCA_000968615.1^23^. The second library was constructed for Hi-C sequencing. It was prepared in manner similar to the Chicago library, with a coverage depth of 1904.26 times of the genome size. The same library preparation protocol was used, and QC was also applied. These libraries prepared by Dovetail Genomics (Scotts Valley, California, USA) were sequenced using an Illumina HiSeq X instrument.

### Genome assembly and downstream analysis

The genome assembly was carried out in two steps. Initially, the Chicago assembly was generated using the Dovetail HiRise pipeline^24^, where the draft assembly (GCA_000968615.1) was used as a reference to map the Chicago reads. The Chicago assembly was then used as a reference to map the Hi-C reads to generate the final genome assembly, again using the Dovetail HiRise pipeline^24^. The assembled genome was also compared against the draft genome (GCA_000968615.1) to check for improvements in the overall quality of the assembly. The genome assembly was then annotated using FunAnnotate^25^, a fungal genome annotation pipeline that identifies protein-coding genes in a fungal genome assembly. First, repetitive contigs were cleaned from the genome for using minimap2^26^. Next, the genome was masked for repeats using RepeatMasker^27^, and Repbase (v20170127)^28^ as the reference database for repetitive elements. FunAnnotate was first run in training mode to improve gene prediction using RNA-seq data from the closely related *T. paradoxa* (SRR15533162)^29^. Then, FunAnnotate was run in prediction mode using the transcriptome of *T. paradoxa* (SRR15533162) assembled with Trinity^30^, a list of Expressed Sequence Tags (ESTs) collected from the National Center for Biotechnology Information (NCBI) using Taxonomy ID: 60496^31^ via Entrez E-utilities^32^, and a list of related protein sequences retrieved from Uniprot^33^. The predicted gene models subjected to the FunAnnotate used InterProScan^34^, Eggnog-mapper^35,36^, and antiSMASH^37^ for functional annotation. In addition, FunAnnotate employed SignalP^38^ to predict the secretome,  and HMMer^39^ to map protein models against dbCAN^40^ for predicting carbohydrate-active enzymes (CAZymes), and diamond^41^ blastp search of MEROPS^42^ database for peptidases prediction.

### Assessment of completeness and continuity of genome assembly

For assembly continuity comparison, genome sequences along with annotations of five *Thielaviopsis* strains: *T. ethacetica* (BCFY00000000.1)^43^, *T. populi* (JADILG000000000.1)^44^, *T. cerberus* (JACYXV000000000.1)^45^, *T. euricoi* (BCHJ00000000.1)^46^, and *T. musarum* (LKBB00000000.1)^47^ were downloaded from the NCBI database. These strains were compared against the newly sequenced *Tp* DSM102798 genome using the sequence length of each assembly with the average scaffold length. The completeness analysis was performed by comparing the results of BUSCO analysis of each genome against fungi_odb10 lineage-specific profile^48^.

## Data Records

All sequence data, including raw Chicago reads and Hi-C short reads, were deposited to the NCBI database under BioProject PRJNA1060910 with accessions SRR27421216^49^ and SRR27421217^50^, respectively. The genome assembly is available through NCBI GenBank with accession JAYKOR000000000^51^. The genome annotation information was deposited in the Figshare database^52^.

## Technical Validation

### Genome assembly

**Fig. 1:**
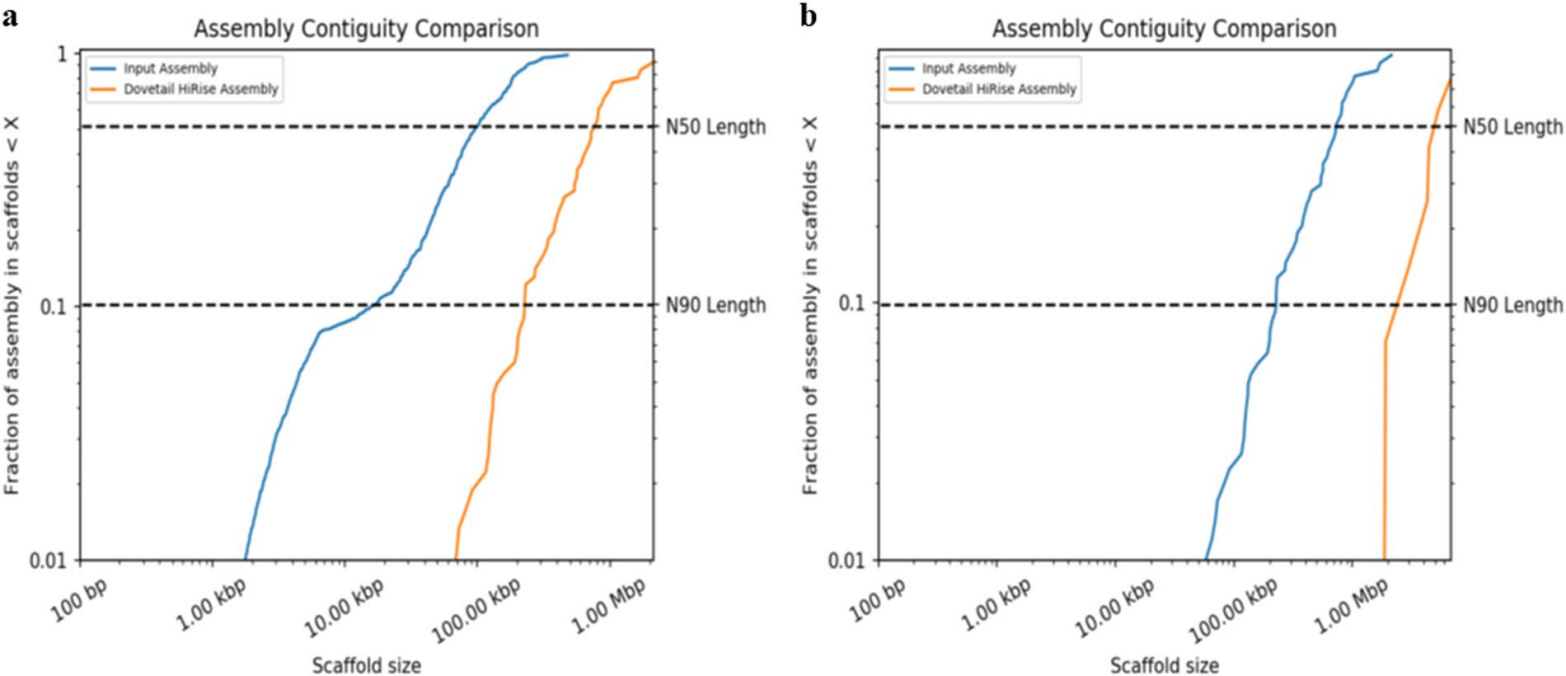
Comparison of the contiguity of the input assembly and final HiRise assembly of *Thielaviopsis punctulata* DSM102798 genome. (**a**) Chicago assembly was first scaffolded with an estimated physical coverage (1–100 kb) that was 1,472.25X; and (**b**) Hi-C assembly which was further improved with estimated physical coverage (10–10,000 kb) of 77,155.15X. Each curve shows the fraction of the total length of the assembly present in scaffolds of a given size or smaller. The fraction of the assembly (scaffolds) is presented on the Y-axis, whereas the scaffold length (bp) is provided on the X-axis. The two dashed lines indicate N50 and N90 lengths of each assembly. Scaffolds less than 1 kb were excluded from the analysis.

**Fig. 2:**
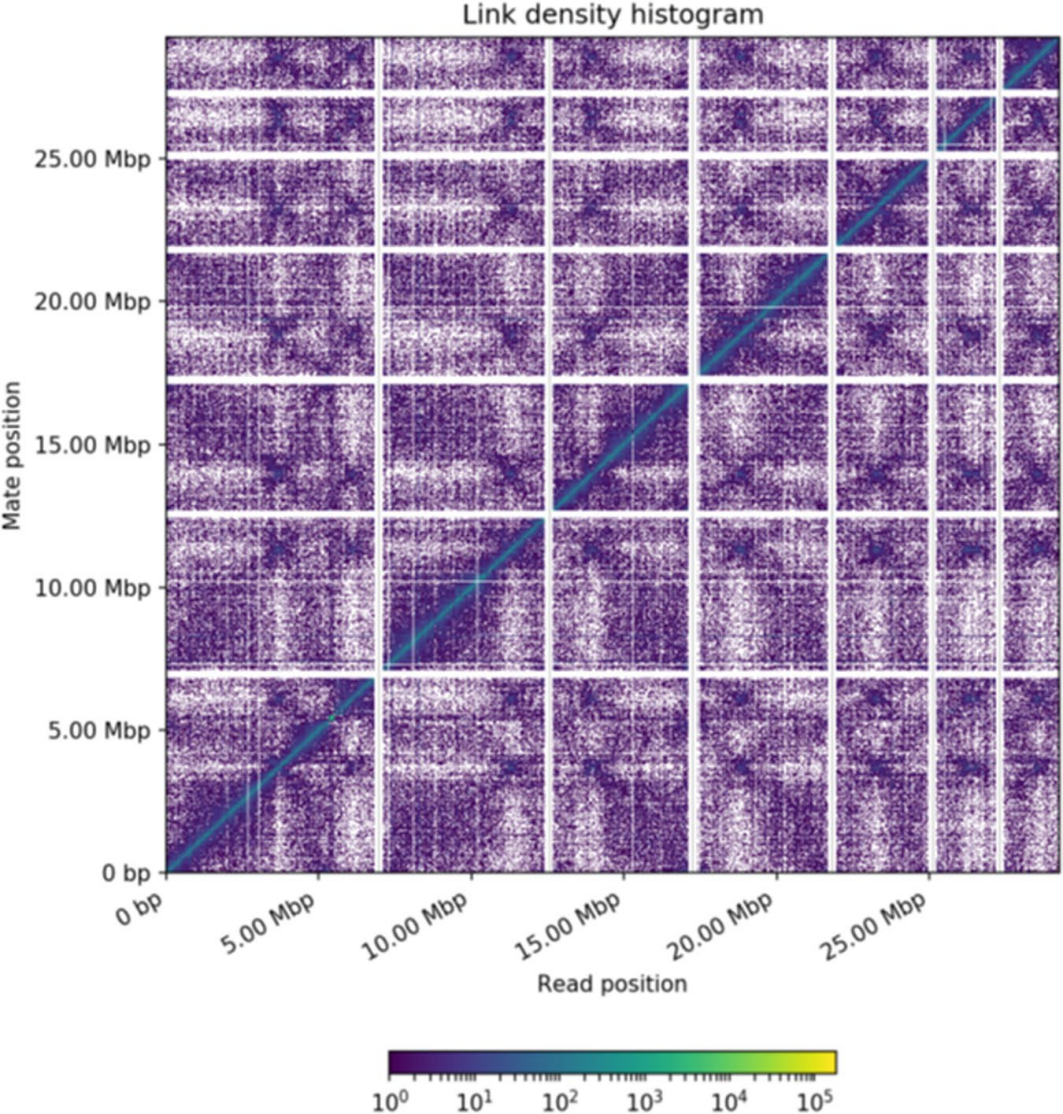
Link density histogram mapped with Hi-C reads. X- and Y-axes represent the mapping positions of the first and second read in each read pair groups, respectively, into bins. For each square container, the color indicates the number of read pairs within the bin. White (vertical) and black (horizontal) lines are provided to show borders between scaffolds. Scaffolds less than 1 Mb were excluded from the analysis.

The Chicago library generated 208 M read pairs (2 × 150 bp) was used to create the primary Chicago assembly using the publicly available genome assembly of *Tp* GCA_000968615.1 as the reference. This produced a Dovetail HiRise assembly of 28.22 Mb with larger scaffolds than GCA_000968615.1 (Fig. 1a). During the assembly process, the HiRise pipeline made 55 breaks and 1,055 joins in GCA_000968615.1. The Chicago assembly then served as a reference to generate the Hi-C assembly against the Hi-C library of 179 M read pairs (2 × 150 bp), where the overall scaffold size was significantly improved due to 60 scaffolds being joined by the HiRise pipeline (Fig. 1b). At the basic level, the quality of the final Hi-C assembly was significantly better than GCA_000968615.1 assembly based on various factors such as scaffold length, N50, N90, and the total number of scaffolds (Table 1). Hi-C contact maps were created from the output of HiRise using Juicer^53^, and the contact map was configured to identify Topologically Associated Domains and A/B genome compartments. The configured contact map was visualised using Juicebox^54^, which revealed seven scaffolds, and made up the genome of *Tp* DSM102798 (Fig. 2).

**Table 1:** A comparison of the reference genome GCA_000968615.1 against Chicago and Hi-C assemblies of *Thielaviopsis punctulata* DSM102798 genome.

Information	GCA_000968615.1	Chicago assembly	Hi-C assembly
Total Length (Mb)	28.12	28.22	28.23
L50/N50 (scaffolds/Mb)	87/0.093	13/0.722	3/4.429
N90 (scaffolds/Mb)	347/0.009	41/0.202	6/1.908
Longest scaffold (bp)	479,719	2,138,895	6,806,703
Number of scaffolds	2,314	1,314	1,242
Contig N50 (kb)	18.91	18.56	18.56
Number of gaps	1,436	2,491	2,551
Percent of genome in gaps (%)	0.78	1.15	1.17

### Genome annotation

**Fig. 3:**
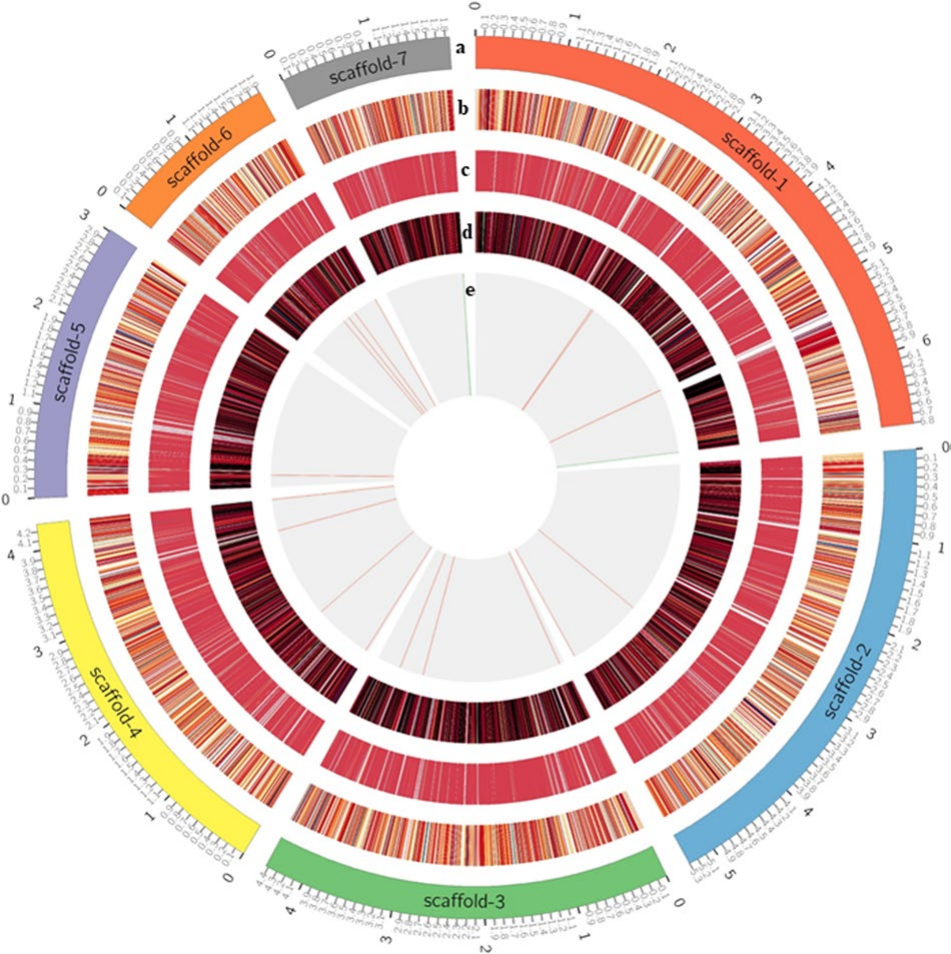
Circos plot of *Thielaviopsis punctulata* DSM102798 genome assembly. (**a**) The seven longest scaffolds of the genome assembly, (**b**) gene density, (**c**) exon density, (**d**) mRNA density and (**e**) long terminal repeats (LTR) density of each scaffold.

**Fig. 4:**
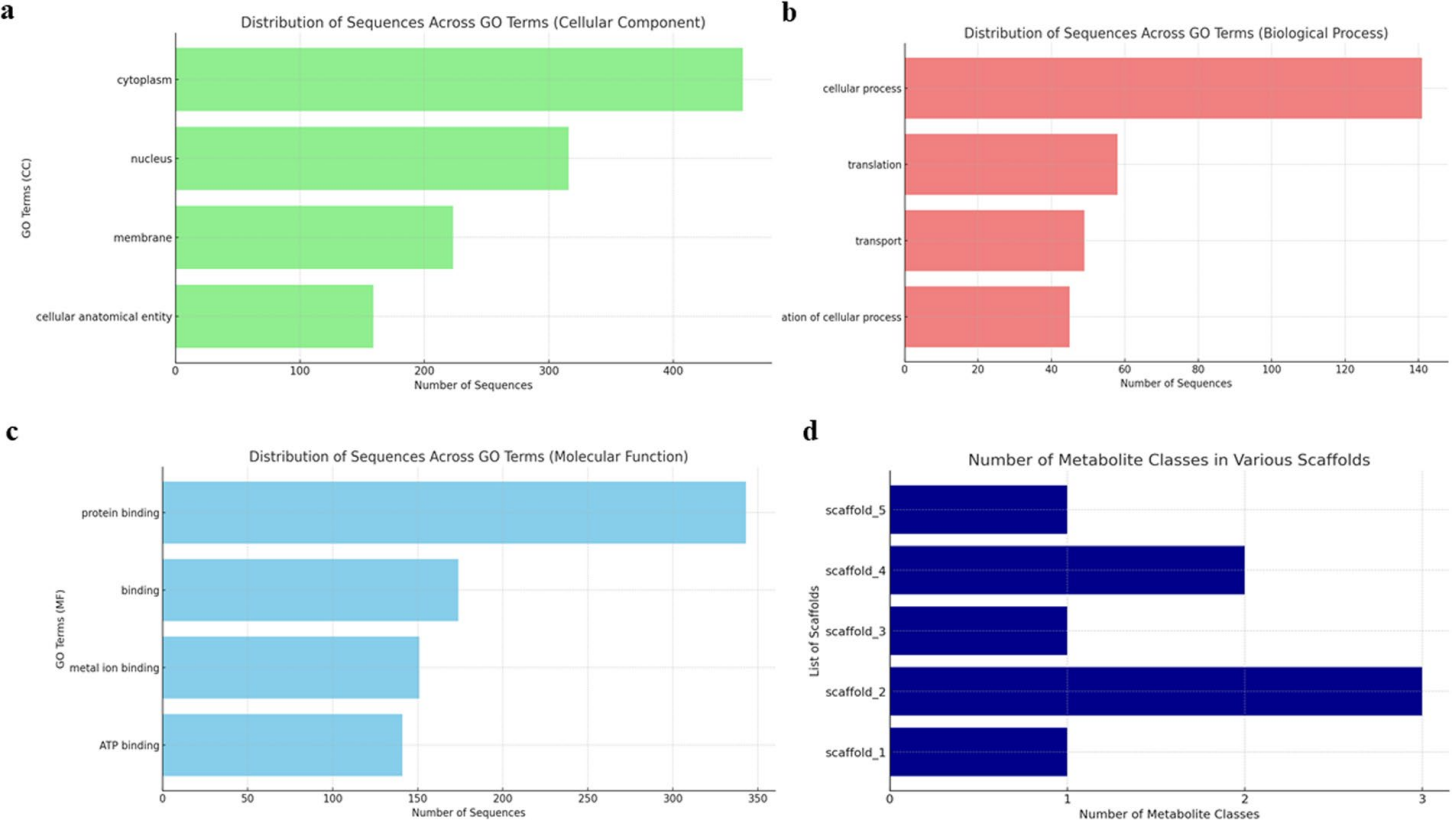
Functional annotation and Gene Ontology (GO) for *Thielaviopsis punctulata* DSM102798. Distribution of sequences for (**a**) cellular components, (**b**) biological process, (**c**) molecular function, and (**d**) number of secondary metabolite biosynthesis gene clusters identified from the first 7 scaffolds of *Tp* DSM102798 genome.

The annotation of Hi-C genome assembly using FunAnnotate predicted 7,169 genes and 18,306 exon sequences; thus, providing important information about the function, structure, and location of genes and other biologically significant elements (Table 2; Fig. 3). GO analysis was carried out using Blast2GO^55^ and eggNOG, yielding 3,501 sequences with 33,829 annotations. There were 1,100 clusters of orthologous genes related to information storage and processing, 1,190 to cellular processes and signaling, and 1,473 to metabolism. GO terms were further categorized based on cellular components (Fig. 4a), biological processes (Fig. 4b), and molecular function (Fig. 4c). The orthologous group distribution revealed that out of 7,169 genes, 6,451 were predicted to be in Kingdom Fungi, 6,438 were specific to Division Ascomycota, and 6,154 belonged to Class Sordariomycetes which perfectly correspond to the taxonomy of *Tp*^30^.

**Table 2:** Summary of gene prediction and genome annotation of *Thielaviopsis punctulata* DSM102798 using FunAnnotate pipeline*.

Attribute	Values
Number of total genes	7,169
Protein length (avg)	522.32
Number of total mRNA	6,974
Number of total tRNA	195
Gene length (avg)	1,735.38
Number of exons	18,306
Exon length (avg)	543.21
GO terms	3,501
Protein models with InterPro hits	5,533
Protein models with EggNOG DB hits	6,482
Protein models with Pfam domains	5,021
CAZymes	226
MEROPS	224
BUSCO (groups)	1,275
Secretion	492

*FunAnnotate uses predicted protein models to perform functional annotation through several tools, including InterProScan, Eggnog-mapper, antiSMASH, and SignalP. It also conducts alignment-based annotation using reference databases such as Pfam 35.0, Uniprot 2022_05, MEROPS 12.0, dbCAN 11.0, MIBiG 1.4, and interPro 92.0. These databases help identify Pfam domains, CAZymes, secreted proteins, peptidases (MEROPS), and BUSCO groups.

Secondary metabolite biosynthesis gene clusters were identified from scaffolds 1–5 of *Tp* DSM102798 genome (Fig. 4d). Dimethylcoprogen has been identified as a siderophore produced by many pathogenic fungi to conquer the battle for iron acquisition^56^. In addition, The complex class of fungal metabolites, squalestatin S1 (zaragozic acid), which Is an inhibitor of squalene synthase that controls the use of cholesterol biosynthesis^57^ was also among the gene clusters of *Tp*.

**Fig. 5:**
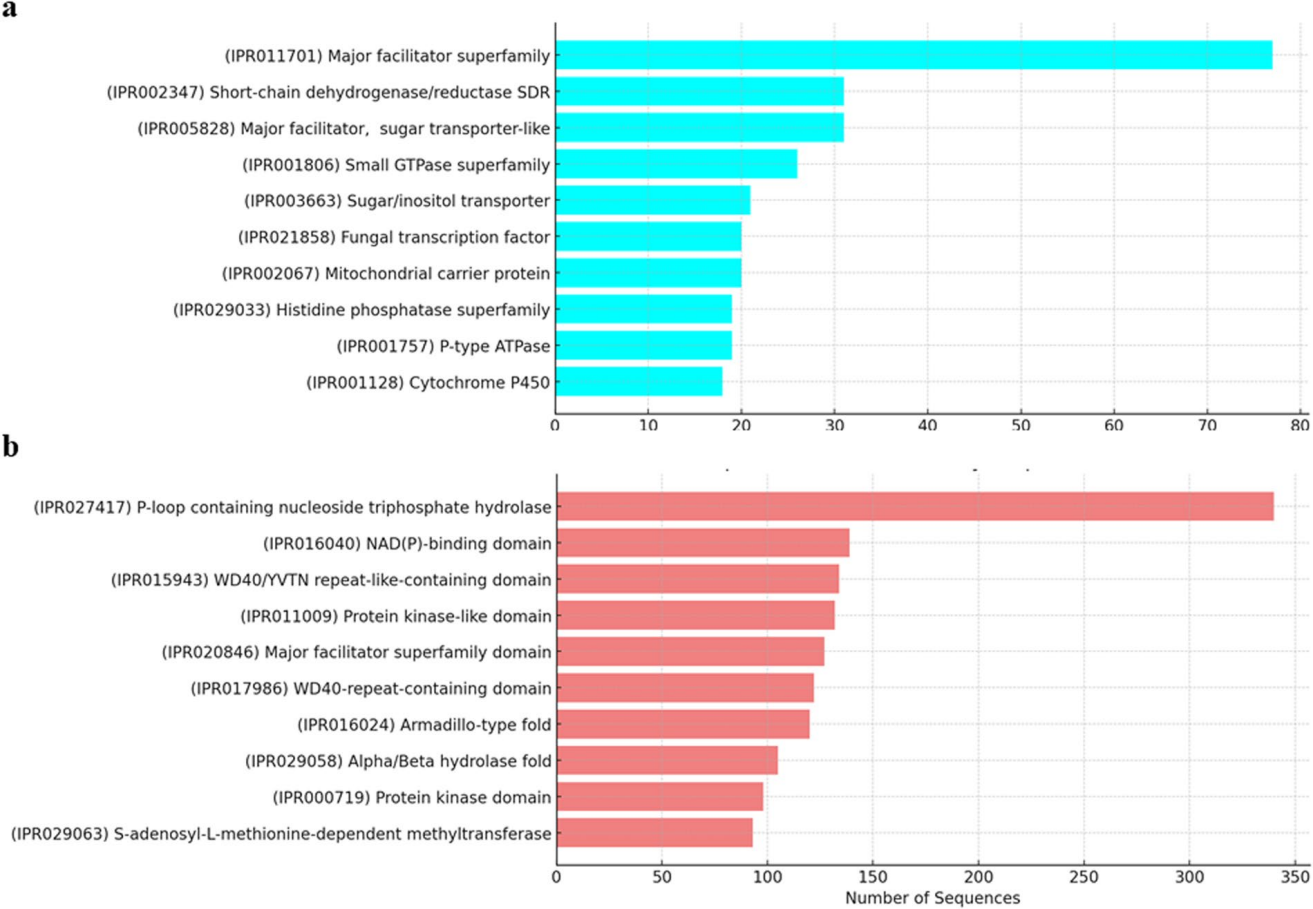
Prediction of protein-related genes identified in *Thielaviopsis punctulata* DSM102798 genome. Top 10 protein (**a**) families and (**b**) domains by sequence count. In (a & b), the top 10 identified protein families and domains with their respective sequence counts were presented.

In addition, 6811 protein families and domains were identified from the genome, including major facilitator superfamily, fungal transcription factor, and cytochrome P450 (Fig. 5a). These superfamily proteins play a significant role in various biological processes such as transporting small solutes across cell membranes and metabolism of drugs and synthesis of cholesterol, steroids, and other lipids. Notable protein domains, such as α/ß-hydrolases, kinase domains and S-adenosyl-L-methionine-dependent methyltransferases that were associated with specific biochemical activities includung enzyme catalysis, substrate binding, and molecular interactions were identified (Fig. 5b).

### Genome continuity and completeness analysis

**Fig. 6:**
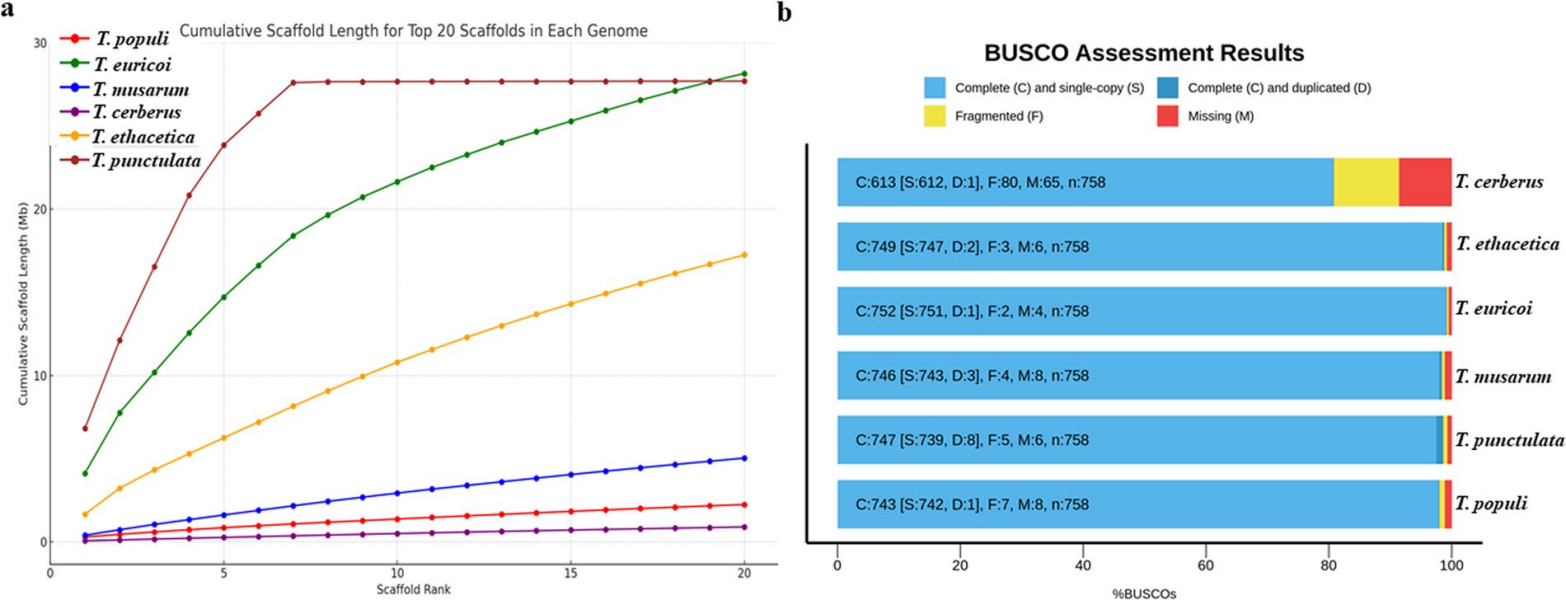
Comparisons in genome assembly of *Thielaviopsis punctulata* DSM102798 and the genomes of other *Thielaviopsis* species. (**a**) Contiguity of the genomes of five *Thielaviopsis* spp. compared to Hi-C genome *Tp* DSM102798 based on the first 20 longest scaffolds from each genome. (**b**) Completeness of the genome assembly of *Tp* DSM102798 compared to that of five other *Thielaviopsis* genomes collected from NCBI database.

Our analysis revealed that *Tp* DSM102798 exhibited the highest continuity among the five *Thielaviopsis* genomes. The cumulative size of the first seven scaffolds/contigs was 27.598 Mb, which surpassed the assemblies of all other *Thielaviopsis* strains, ranging from 0.360 Mb in *T. cerberus* to 18.391 Mb in *T. euricoi* (Fig. 6a). The same genomes were compared for their completeness using BUSCO, and *Tp* DSM102798 also achieved a completeness rate of 98.6% for the 758 orthologous genes in the Fungi_odb10 database (Fig. 6b).

## Data Availability

This work did not utilise a custom script. Data processing was carried out using the protocols and manuals of the relevant bioinformatics software.
